# Impact of pharmacological and psychological treatment methods of depressive and anxiety disorders on cognitive functioning

**DOI:** 10.1007/s00702-014-1282-3

**Published:** 2014-07-31

**Authors:** Krzysztof Krysta, Marek Krzystanek, Małgorzata Janas-Kozik, Adam Klasik, Irena Krupka-Matuszczyk

**Affiliations:** 1 Department of Psychiatry and Psychotherapy, Medical University of Silesia, ul. Ziołowa 45/47, 60-635 Katowice, Poland; 2 Department of Child and Adolescent Psychiatry and Psychotherapy, Medical University of Silesia, ul. Zapolskiej 3, 41-218 Sosnowiec, Poland; 3 Institute of Psychology, University of Opole, Plac Staszica 1, 45-052 Opole, Poland

**Keywords:** Pharmacotherapy, Psychotherapy, Depression, Anxiety, Cognitive functions

## Abstract

Anxiety and depressive disorders are characterized by a number of clinical symptoms like decreased mood, apathy, anhedonia and anxiety. An important element of the clinical picture is also neurocognitive impairment. The most common treatment methods for depression and anxiety are pharmacology, psychotherapy or a combination of both methods. The data from literature show that those treatment methods lead to an improvement of clinical symptoms, but they exert a possible impact on cognitive functions. However the study results referring both to the role of pharmacological treatment and psychotherapy in this domain are still inconsistent. There is an increasing number of accessible data confirming the positive effects of those clinical interventions on cognitive functioning of anxiety and depressive patients, but the interpretation is complicated because of differences in methodology as well as examined sample size and their characteristics. More studies are then needed to describe this phenomenon.

## Introduction

Cognitive impairment is an important element of the clinical picture of psychiatric diseases which requires appropriate treatment and rehabilitation. According to neuropsychological literature it is defined as a decline in function in one or more domains of cognitive function. It may represent a single deficit which is highly specific or a cluster of deficits related to each other (Lezak et al. 2004). It is being discussed by experts, what should be the role of cognitive deficits in the diagnostic systems, and how they can discriminate such disorders like schizophrenia and affective disorders (Bora et al. 2010).

## Cognitive deficits in depression

Traditional studies of unipolar depression and anxiety disorders focus on typical symptoms like decreased mood, apathy, anhedonia and anxiety but they do not pay sufficient attention to the cognitive functioning of the patients. Cognitive impairment may produce serious alteration in daily functioning. Cognitive deficits in major depressive disorder (MDD) in such domains as working memory, attention, and psychomotor processing speed are consistent, replicable, nonspecific, and clinically significant. They are regarded to be responsible for psychosocial impairment (McIntyre et al. 2013). Deficits in memory and decision-making are supposed to be present early in the course of the disease (Trivedi and Greer 2014). They are observed already in the first episode. Psychomotor speed and memory functioning are found to be associated with clinical state. Attention and deficits in executive functioning are regarded to be trait elements of MDD (Lee et al. 2012). In studies referring to euthymic depressive patients it was found that certain cognitive deficits like verbal memory, speed of information processing and some executive functions are persistent even during remissions (Bora et al. 2013). The study of Schaub et al. (2013) suggests that the most common deficits in this domain present in depressed patients relate to verbal and visual short-term memory, verbal fluency, visual-motor coordination, information processing in visual-verbal functioning and selective attention, however the results were not compared with healthy controls. Problems with attention and memory refer to the diagnostic criteria of depression and the patient’s stopping to meet the criteria of depressive episode does not imply the cognitive symptoms to disappear. It was summarized by Roiser and Shakian (2013) that cognitive impairment non-related to emotional state is present in unipolar depression patients and that it tends to maintain even after the remission from a depressive episode. Moreover, the preserved cognitive impairment predicts poor response to the treatment with antidepressants. The relation between cognitive impairment in depression and the disease itself is not established. As reviewed by Goeldner et al. (2013), the cognitive deficit in depression seems incorporated in the body of depressive process, however in some subsets of the patients suffering from MDD, the intensity of cognitive impairment does not relate to the severity of depressive symptoms. The cognitive impairment is also present in patients who maintain residual symptoms of depression like fatigue, apathy and feeling a little low mood. The study of Pedrelli et al. (2010) showed that fatigue and feeling “blue” correlate with preservation of specific cognitive problems like difficulties in concentration and alertness. In the study of Gualtieri et al. (2006) the cognitive impairment, occurring during the depressive episode improved after the treatment with antidepressive drug but was not normalized. Apart from the concept of the two separated notions, different neuropsychlogical and some new neuroimagining studies (e.g. Pu et al. 2011) accumulate the evidence of the biological link between depression and the impairment of the cognition. One of the proposals, explaining problems in cognition among depressive patients is the depression-executive dysfunction (DED) model. According to DED model, the impairment of cognitive functioning, especially the executive functions, is likely to result in the poor response to antidepressants. However, due to the insufficient number of studies the hypothesis cannot be validated so far (McLennan and Mathias 2010).

Another issue raises from the biological treatment of depression. Antidepressants with alpha-adrenergic, anti-cholinergic and anti-histaminic activities or electro-convulsive therapy may cause significant cognitive impairment. However, the impact of medication appears to be mild and with doubtful clinical significance (Gorenstein et al. 2006). The problem is even more relevant for anxiety patients using benzodiazepines. The review done by Stewart (2005) showed that benzodiazepines produce cognitive dysfunctions in patients treated for longer periods and that the cognitive impairment may be preserved after the benzodiazepine withdrawal.

## Cognitive deficits in anxiety disorders

Comparable deficits in this domain are present also in anxiety disorders (Hindmarch 1998). In anxiety disorders, it is proposed that cognitive impairment is a primary symptom, although it is well known that drugs, especially benzodiazepines interfere with cognition, too. The impact of anxiety on cognitive functioning is however less explored than in other disorders including depression (Jakuszkowiak-Wojten et al. 2013). Some early studies of this subject showed even no cognitive deficits in such conditions like panic disorder and social phobia (Gladsjo et al. 1998). However, according to other authors, reduced performance in panic disorder and social phobia patients, relative to control subjects, are found in the domains of verbal learning and memory (Asmundson et al. 1995). An intentional bias toward negatively–valenced verbal stimuli and longer decision-making latencies were observed by the authors of the study performed on subjects suffering from panic disorder (Kaplan et al. 2006). In another research project related to this group of patients, deficits in delayed visual memory and recognition test in CANTAB test were found (Raczak et al. 2013).

## Objective of the analysis

There is an increasing number of data from literature showing that those cognitive deficits present in depressive and anxiety patients may decrease as a result of successful treatment. The objective of our analysis is to find out the impact of different pharmacological and psychological methods of treatment of depressive and anxiety disorders on the cognitive functioning in affected patients. The focus of this review was on studies on depression and anxiety disorders (1990–2014). We searched PubMed using the following search terms [effective date: 12th March 2014): (depression (Title/Abstract) OR anxiety (Title/Abstract)] AND pharmacotherapy (Title/Abstract) OR psychotherapy OR cognitive functions OR memory OR attention OR executive functions AND psychomotor speed AND English (lang) AND (1990/01/01(PDAT): 2014/03/12(PDAT)).

## Impact of pharmacological treatment of depressive and anxiety disorders on cognition

The number of studies referring to the impact of pharmacological treatment of depressive and anxiety disorders on cognition is growing, however they still bring inconsistent results. Table 1.

**Table 1:** Overview of the studies on the impact of pharmacotherapy on cognitive functions

References	Diagnose	No. of the patients	Studied compound	Duration of the study	Cognitive battery
Borkowska et al. (2007)	Major depressive disorder	71	Mirtazapine	6 months	Wisconsin Card Sorting Test, *N*-back test, TMT, Stroop Tests
Braszko et al. (2003)	Depressive symptoms in non-depressive patients	39	Captopril, enalapril	4 weeks	The Rey Auditory Verbal Learning Test, Wechsler Memory Scale
Chang et al. (2012)	Major depressive disorder	149	Fluoxetine, venlafaxine	6 weeks	Continuous Performance Test (CPT), Finger-Tapping Test (FTT), and Wisconsin Card-Sorting Test (WCST)
Constant et al. (2005)	Major depressive disorder	20	Sertraline	7 weeks	Phasic Alertness Task, Stroop Test, The Supraliminal and Subliminal Emotional Stroop,
Galassi et al. 2006	Major depressive disorder	48	Fluoxetine, reboxetine	6 months	Attentional matrices, Wechsler memory scale, Familial faces recognition, Famous faces recognition, Stem completion test, Autobiographical memory, MLT ‘88: test for historical events, MMSE
Gorenstein et al. (2006)	Major depression	42	Imipramine, clomipramine, fluoxetine, sertraline	>6 months (imipramine for 2.4 ± 0.6 years, clomipramine for 2.8 ± 1.2 years, fluoxetine for 1.8 ± 0.3 years, sertraline for 1.5 ± 0.3 years)	Subjective Memory Questionnaire (SMQ), Verbal recall, Word appreciation task, Digit span forward and backward, Word stem completion, Visual recall (from Memory I, PSS), Digit–Symbol Substitution Test (DSST), Cancellation Task (CT), Symbol Copying Test (SCT), Tapping (from Vienna Test System), Inserting pins (from Vienna Test System), Reaction times (from Foundations I, PSS)
Gualtieri et al. (2006)	Major depressive disorder	31	Citalopram, fluoxetine, escitalopram, paroxetine, mirtazepine, trazodone, venlafaxine, bupropion, sertraline	4 weeks	Finger Tapping Test, Symbol-digit Cod, Stroop Test, The Shifting Attention Test, Continuous Performance Test
Han et al. (2011)	Major depression	281	Fluoxetine, sertraline, fluvoxamine, paroxetine and citalopram, amitriptyline, desipramine, doxepine, imipramine, nortriptyline, trimipramine clomipramine, maprotiline, tranylcypromine, trazodone, nefazodone, venlafaxine, bupropion	12 months	Mini-Mental State Examination
Herrera-Guzmán et al. (2009)	Major depressive disorder	73	Escitalopram, duloxetine	24 weeks	WAIS III vocabulary subtest, WAIS III digit span, Rey auditory verbal learning test (RAVLT), Pattern recognition memory (PRM), Spatial recognition memory (SRM), reaction time (RTI)
Herrera-Guzmán et al. (2010)	Major depressive disorder	73	Escitalopram, duloxetine	24 weeks	WAIS III vocabulary subtest, WAIS III Digit span, Spatial Working Memory (SWM), Rapid Visual Information Processing (RVIP), Match to Sample Visual Search (MTS), Stroop Test, Intra–Extra-Dimensional Set Shift (ID/ED), Stockings of Cambridge (SOC)
Klasik et al. (2011)	Major depressive disorder	20	Tianeptine	3 months	Vienna Test System
Levkovitz et al. (2012)	Major depressive disorder	46	SAMe (adjunctive to SRI drug)	6 weeks	Cognitive and physical symptoms questionnaire (CPFQ)
Pedrelli et al. (2010)	Major depression disorder	117	Tricyclic antidepressants, benzodiazepines, mirtazapine	>10 months	CPFQ
Pelton et al. (2008)	Depression (major depressive disorder, dysthymic disorder, or depression not otherwise specified	23	Donepezil	12 weeks	Buschke Selective Reminding Test (targeting memory), WAIS-III digit symbol, Trails B (executive function), CFL (verbal fluency), Trails A (attention/psychomotor speed)
Raskin et al. (2007)	Major depressive disorder	311	Duloxetine	8 weeks	Verbal Learning and Recall Test, the Symbol Digit Substitution Test, the Two-Digit Cancellation Test, the Letter-Number Sequencing Test
Schrijvers et al. (2009)	Major depressive disorder	19	Sertraline	6 weeks	Computed copying tasks, Symbol Digit Substitution, Task (SDST, a subtest of the Wechsler Adult Intelligence Scale)

A relationship between the improvement of cognitive functioning as a result of antidepressant drugs was observed in a group of 50 patients treated with fluoxetine and reboxetine. Partial improvement in attention and memory was achieved, and no differences between patients treated with fluoxetine and reboxetine were found (Galassi et al. 2006). In other studies escitalopram and duloxetine improved the deficits of attention and executive functions (Herrera-Guzmán et al. 2010), as well as memory and mental processing speed (Herrera-Guzmán et al. 2009). Some reports confirm the positive impact of sertraline on cognition in patients with depressive and anxiety disorders (Constant et al. 2005; Schrijvers et al. 2009). In our own study we observed that a 3-month treatment with tianeptine led to the improvement of attention and short-term memory in patients with mild and moderate depression (Klasik et al. 2011). On the other hand, in a 12-month study performed on a group of 281 old depressive patients aged 65 and older, antidepressant effect of citalopram, sertraline or paroxetine was not related to the improvement of cognitive symptoms (Han et al. 2011). Antidepressants were also found non-effective in the treatment of cognitive dysfunctions. The attempts to find any specific intervention to improve the cognitive symptoms in depression are promising. One of the agent is S-adenosyl methionine (SAMe). The naturally existing molecule may hypothetically modulate the neurotransmitter metabolism and improve the cognition in MDD (Krzystanek et al. 2011). In one of the recent studies, SAMe was added to the treatment with serotonin re-uptake inhibitor in 46 MDD non-responders (Levkovitz et al. 2012). It was found that SAMe improves the ability to recall information in the studied group. Talarowska et al. (2012) examined the total antioxidant status (TAS) in depressive patients and she found that the higher the concentration of TAS in plasma of depressive patient is, the higher both the severity of depression and the impairment of short- and long-term declarative memory, working memory and verbal fluency are. Another study of 149 MDD patients revealed that cognitive performance, as well as treatment response in depressive patients, correlate with plasma CRP levels (Chang et al. 2012). Results of such studies may imply that anti-inflammatory drugs, as the adjunctive treatment, may play a role in preservation and/or improvement of cognition in depressive patients. A similar example is the use of minocycline—neuroprotective and anti-inflammatory compound. It was reviewed by Pae et al. (2008) that minocycline may be effective for augmentation of antidepressants in the treatment of cognitive disfunctions in MDD. Another approach to identify the agents, producing specific improvement of cognition in depressive and anxiety patients is a use of sigma-1 receptor agonist. From this point of view, very interesting is the attempt to use fluvoxamine, the potent sigma-1 receptor agonist. Fluvoxamine was proved in animal models and in human studies to improve the cognitive impairments (Hindmarch and Hashimoto 2010). Borkowska et al. (2007) reported the specific pro-cognitive effect of mirtazapine in improving cognitive impairment in unipolar depression. It is worth noticing that the improvement of cognition does not correlate with recovery from depressive symptoms and it occurred after 3 months of the mirtazapine treatment. After 6 months of the treatment, patients achieved the cognitive test results comparable with healthy controls. In one study also donepezil was found to improve the cognitive impairment in depressive patients aged 50 or more (Pelton et al. 2008). Donepezil in this study was added to the ongoing treatment with antidepressant. Raskin et al. (2007) studied the influence of duloxetine on cognitive deficits in MDD elderly patients. The study was done in considerable number of patients and revealed that duloxetine can significantly improve memory and verbal learning. Another small sample study with captopril and enalapril, angiotensin converting enzyme inhibitors, showed the antihypertensive drugs can improve cognition in non-depressive subjects (Braszko et al. 2003).

It is too early to draw any conclusions from the studies on the relation between the antidepressants and cognitive impairment in unipolar depression. Especially in anxiety disorders the studies are less than few and the field requires further exploration. Many more studies are necessary to establish the real impact of antidepressants on cognition, as well as to discriminate the negative influence of some antidepressants on cognition and to create new drug strategies for improvement of the cognitive impairment in depression and anxiety.

## Impact of psychological treatment methods of cognitive impairment in depression and anxiety disorders

Except for pharmacology there is a number of non-biological interventions which may directly or indirectly lead to cognitive improvement in depressed patients. One group are interventions, whose primary goal is a cognitive training as a cognitive remediation. And the other group includes the traditional methods of psychotherapy, like psychodynamic psychotherapy, CBT, interpersonal psychotherapy, for which the main goal is the improvement of patient`s clinical symptoms. The impact on neurocognition may be an additional value of the therapeutic interventions.

## Role of cognitive training methods

The usefulness of the cognitive remediation was confirmed in a number of studies. Elgamal et al. (2007) observed a positive impact of cognitive training on attention, verbal learning and memory, psychomotor speed and executive function in recurrent major depressive disorder. Naismith et al. (2010) observed improvement in memory performance in patients with affective disorders. Bowie et al. (2013a) confirmed a positive impact of daily online cognitive exercises on such domains like attention/processing speed and verbal memory in patients with treatment-resistant depression. The authors of the above study analyze and emphasize the advantages on cognitive remediation trainings in mood disorders in their review (Bowie et al. 2013b).

## Neuroimaging studies evidence of the psychotherapy effectiveness in cognitive improvement

### Depressive disorders

In literature we can find an increasing number of studies analyzing the neurobiological basis of psychotherapy (de Vries et al. 2013), however the research data are still not consistent and valuable, as the number of studies is still small and they differ in methodology and evaluated treatment strategies (Karch et al. 2012). Some of the adopted methods of analyzing the impact of psychotherapy on brain functioning are neuroimaging techniques. Modulation of limbic and cortical regions was observed in patients with unipolar depression treated with CBT (Goldapple et al. 2004). In some studies certain differences in the effect of both treatment methods are reported. The study comparing the results of the treatment with CBT and venlafaxine in a group of patients with major depressive disorder, revealed that CBT modulates cortical-limbic connectivity and that venlafaxine engages additional cortical and striatal regions (Kennedy et al. 2007).

### Anxiety disorders

It was found that cognitive-behavioral therapy of panic disorder reduces the hyperactivity in several brain areas causing adaptive metabolic changes of the medial prefrontal cortex (Sakai et al. 2006). Similar changes were found after the treatment either with CBT or with antidepressants in patients suffering from panic disorder (Prasko et al. 2004) or from social phobia (Furmark et al. 2002).

### Importance of methodological limitations

The field of neuroimaging studies confirming the effects of psychotherapy on modulation of functioning on different structures of the brain is still growing, bringing more and more findings (Lindauer et al. 2008; Buchheim et al. 2012; Cervenka et al. 2012; Gawrysiak et al. 2013; Goldin et al. 2013; Morgiève et al. 2013; Schiepek et al. 2013) Recent advances in this filed were reviewed by Barsaglini et al. 2013. The authors conclude that the impact of psychotherapy on measurable changes in the brain is beyond doubt, but it appears to be dependent on the investigated disorder and the type of the therapy used. Messina et al. (2013) in their meta-analysis also confirm positive neural changes in brain networks associated to emotion regulation after psychotherapy of depression and anxiety drawing the attention to methodological limitations like small sizes, lack of suitable control groups, heterogeneity of techniques or study designs.

### Neuropsychological studies evidence of the beneficial effect of psychotherapy on cognition

Contrary to the results of neuroimaging studies, the access to research data related to the neuropsychological evaluation of cognitive changes occurring in the process of psychotherapy is very limited. This also refers to studies comparing the cognitive functioning in patients treated with psychotherapy and pharmacotherapy. Table 2.

**Table 2:** Overview of the studies on the impact of psychotherapy on cognitive functions

	Diagnose	No. of patients	Duration of the study	Cognitive battery	Model of psychotherapy	Additional pharmacotherapy
Klasik et al. (2012)	Recurrent Depressive Disorder	60	8 weeks	Vienna Test System (VTS)	Psychodynamic	Sertraline (in 2 of 3 examined groups)
Bastos et al. (2013)	Moderate depression	272	2 years	WAIS-III	Psychodynamic	Fluoxetine (in 2 of 3 examined groups)
Reinecke et al. (2013)	Panic disorder	28	4 weeks	Attention visual probe task	CBT	Exclusion criterion: psychopharmacological treatment during the last 6 months
Dobkin et al. (2014)	Major Depressive Disorder (MDD), Dysthymia, or Depression Not Otherwise Specified, Parkinson`s disease	80	10 weeks	Hopkins Verbal Learning Test–Revised (HVLT–R), Delis-Kaplan Executive Function System (D–KEFS), Wechsler Memory Scales, Stroop Color and Word Test	CBT	Stable medication regimen for ≥6 weeks
Mackin et al. 2013	Late life depression	221	24 weeks	Stroop Color and Word Test, Hopkins Verbal Learning Test - Revised (HVLT–R), DRS-IP, Wisconsin Card Sorting Test-64 Computer Version, Trail Making Test (TMT)	Problem Solving Therapy, Supportive Therapy	Antidepressant-free participants

In a recent study performed in Brasil 272 patients aged 26–34 were examined. They were randomly divided in three subgroups. The first subgroup was treated with long-term psychodynamic psychotherapy, the second with fluoxetine, and the third received both psychotherapeutic and pharmacological treatment. The intensity of depression was evaluated with the use of Beck Depression Inventory (BDI), and the neurocognitive deficits assessed with the Wechsler adult intelligence scale, third edition (WAIS-III). The study lasted 2 years, and the neuropsychological examination was repeated every six months. Patients treated with the combination of psychodynamic psychotherapy and fluoxetine presented the greatest improvement in cognitive scores at the end of the study. The lowest improvement was observed in the group of patients treated with fluoxetine (Bastos et al. 2013). The results of this study were similar to our own. However, the number of patients participating in our research project was smaller, we recruited 60 patients with a diagnosis of recurrent depressive disorder. They were also divided into three subgroups treated with pharmacotherapy with sertraline, psychodynamic psychotherapy or both. The neuropsychological changes occurring during the treatment process were assessed with the Vienna Test System (VTS). In comparison to the Brasilian study, the time of observation was much shorter (8 weeks). The improvement in short-term memory and attention was also the most prominent in patients treated with both forms of therapy. However, contrary to the results of the Brasilian study, the worst performing group consisted of patients was treated with psychotherapy alone (Klasik et al. 2012). What is the mechanism of information processing during the psychodynamic therapeutic process, still remains unclear. Novac (2013) proposed the use of a term “transprocessing” illustrating the discussed phenomena. This term is a combination of two terms. The first term is transduction, which is reexpression and translation of a functional brain activity into a new type activity, like transduction of stress in the function of the hypothalamopituitary axis. The second term is processing, a function by which the brain makes sense of environmental inputs. According to the author, changes in these processes during psychotherapy are internalized and assimilated. A small number of studies analyze the influence of Cognitive-Behavioral Therapy (CBT) on neurocognition. Reinecke et al. (2013) performed a study on a group of 28 patients with panic disorder. Attention visual probe task was performed a day after the treatment session and 4 weeks later. The authors observed the decrease of vigilance for threat information in the treated group, and the magnitude of this early effect predicted therapeutic response. There is also evidence from literature that psychotherapy may improve neurocognition in depressive disorders related to a psychoorganic condition. Mackin et al. (2013) observed a beneficial effect of psychotherapy on cognitive functioning after psychotherapy treatment of depression in older adults with executive dysfunction. Dobkin et al. (2014) described a neuropsychological improvement in patients with Parkinson’s disease after cognitive-behavioral treatment of depression. Improvements in verbal memory and executive functioning were observed after 10-week treatment period. Collerton (2013) proposed the explanation of possible structural and functional brain changes after CBT psychotherapy treatment related to neuroplasticity in the linked systems of the frontal, cingulate, and limbic cortex. Except from the theories aiming at the explanation of psychotherapy effects, efforts are also made to analyze the interaction of combined pharmacological and psychological interventions. An interesting approach was presented by Castrén (2013), proposing a hypothesis that antidepressant agents reactivate juvenile-like plasticity in the cortex, which facilitates further recovery of neuronal networks related to psychotherapy and other non-pharmacological interventions in mood disorders.

## Conclusions

Deficits in neurocognition play a significant role among other symptoms of depressive and anxiety disorders. It is important for the clinicians to what extent the commonly used methods of treatment of these conditions exert a positive influence on cognitive processes. The results of studies using pharmacological treatment are inconsistent. There is a group of research findings suggesting a favorable effect of antidepressive treatment on this domain of symptoms (Galassi et al. 2006; Borkowska et al. (2007); Herrera-Guzmán et al. 2009, 2010; Klasik et al. 2011). Other data from literature suggest no cognitive improvement following treatment of antidepressants (Han et al. 2011). Efforts are made to introduce other types of pharmacotherapy in order to reach the expected goal, like the use of donepezil (Pelton et al. 2008) or captopril and enalapril (Braszko et al. 2003), S-adenosyl methionine (SAMe) (Krzystanek et al. 2011; Levkovitz et al. 2012). Anyway, the studies on the impact of antidepressant medication on cognitive functioning in depression and anxiety patients are still insufficient in number and they do not allow to come to any final conclusion about its effectiveness towards the cognitive impairment in depression. On the other hand, an increasing interest of the researches is focused on the impact of non-biological treatment methods on this domain of symptoms. This interest is enhanced by a growing data from neuroimaging studies confirming the impact of functional changes in brain structures following psychotherapeutic process (Barsaglini et al. 2013; Messina et al. 2013). Some reports confirm the positive effect of different psychotherapeutic treatment methods on the improvement of cognitive skills alongside with clinical improvement (Klasik et al. 2012; Bastos et al. 2013; Reinecke et al. 2013; Dobkin et al. 2014). The above studies are small in number and do not allow to draw unequivocal conclusions. What is however suggested by research study results in this field, psychotherapy and pharmacotherapy used in combination may be more effective than each of those methods used alone (Klasik et al. 2012; Bastos et al. 2013, Castrén 2013).

The above analysis suggests that the commonly used pharmacological and non-pharmacological treatment strategies targeted to help patients suffering from depressive and anxiety disorders may have a positive influence non only on clinical symptoms of those conditions, but also on patients` cognitive functioning. Because of a relatively small number of studies and different methodological attitudes, we still need to continue our research in order to explain discussed phenomenon more profoundly. Except for the widely used treatment methods like pharmacotherapy and psychotherapy, additional techniques of cognitive enhancement should be used in the treatment process, both biological, like e.g. transcranial magnetic stimulation techniques (Balconi 2013), and non-biological, like cognitive training (Elgamal et al. 2007; Naismith et al. 2010; Bowie et al. 2013a).
